# Clinical and Economic Impact of COVID-19 on Agricultural Workers, Guatemala^1^

**DOI:** 10.3201/eid2813.212303

**Published:** 2022-12

**Authors:** Daniel Olson, Diva M. Calvimontes, Molly M. Lamb, Gerber Guzman, Edgar Barrios, Andrea Chacon, Neudy Rojop, Kareen Arias, Melissa Gomez, Guillermo A. Bolanos, Jose Monzon, Anna N. Chard, Chelsea Iwamoto, Lindsey M. Duca, Nga Vuong, Melissa Fineman, Kelsey Lesteberg, David Beckham, Mario L. Santiago, Kendra Quicke, Gregory Ebel, Emily Zielinski Gutierrez, Eduardo Azziz-Baumgartner, Frederick G. Hayden, Hani Mansour, Kathryn Edwards, Lee S. Newman, Edwin J. Asturias

**Affiliations:** Fundacion para la Salud Integral de los Guatemaltecos, Retalhuleu, Guatemala (D. Olson, D.M. Calvimontes, G. Guzman, E. Barrios, A. Chacon, N. Rojop, K. Arias, M. Gomez, G.A. Bolanos, E.J. Asturias);; University of Colorado School of Public Health, Aurora, Colorado, USA (D. Olson, M.M. Lamb, M. Fineman, L.S. Newman, E.J. Asturias);; University of Colorado School of Medicine, Aurora (D. Olson, K. Lesteberg, D. Beckham, M.L. Santiago, L.S. Newman, E.J. Asturias);; La Comisión Presidencial de Atención a la Emergencia COVID-19, Guatemala City, Guatemala (D.M. Calvimontes, E.J. Asturias);; Centers for Disease Control and Prevention, Guatemala City (J. Monzon, E. Zielinski Gutierrez);; Centers for Disease Control and Prevention, Atlanta, Georgia, USA (A.N. Chard, C. Iwamoto, L.M. Duca, N. Vuong, E. Azizz-Baumgartner);; Colorado State University, Fort Collins, Colorado, USA (K. Quicke, G. Ebel);; University of Virginia School of Medicine, Charlottesville, Virginia, USA (F.G. Hayden);; University of Colorado, Denver, Colorado, USA (H. Mansour);; Vanderbilt University School of Medicine, Nashville, Tennessee, USA (K. Edwards)

**Keywords:** COVID-19, coronavirus disease, severe acute respiratory syndrome coronavirus 2, SARS-CoV-2, coronaviruses, viruses, respiratory infections, agricultural workers, severity, cohort, clinical impact, economic impact, respiratory illness impact, productivity, respiratory syncytial virus, influenza virus, zoonoses, Guatemala

## Abstract

We evaluated clinical and socioeconomic burdens of respiratory disease in banana farm workers in Guatemala. We offered all eligible workers enrollment during June 15–December 30, 2020, and annually, then tracked them for influenza-like illnesses (ILI) through self-reporting to study nurses, sentinel surveillance at health posts, and absenteeism. Workers who had ILI submitted nasopharyngeal swab specimens for testing for influenza virus, respiratory syncytial virus, and SARS-CoV-2, then completed surveys at days 0, 7, and 28. Through October 10, 2021, a total of 1,833 workers reported 169 ILIs (12.0 cases/100 person-years), and 43 (25.4%) were laboratory-confirmed infections with SARS-CoV-2 (3.1 cases/100 person-years). Workers who had SARS-CoV-2‒positive ILIs reported more frequent anosmia, dysgeusia, difficulty concentrating, and irritability and worse clinical and well-being severity scores than workers who had test result‒negative ILIs. Workers who had positive results also had greater absenteeism and lost income. These results support prioritization of farm workers in Guatemala for COVID-19 vaccination.

Essential workers have been at greater risk for COVID-19 than for the general population, but little is known about the risk to persons working within the agricultural sector in low- to middle-income countries (LMICs) (*1*–*3*). Limited data from the United States have demonstrated a high burden of SARS-CoV-2 in this population (*1*); many agricultural workers continued working throughout the pandemic (*4*). In LMICs, agricultural workers play a critical role in food security and represent a major economic force. In Guatemala, these workers are 35% of the overall labor force, and agricultural products account for 45% of all exports and 11.3% of total gross domestic product (*5*). Guatemala is also a major trading partner of the United States, exporting US $2.1 billion in agricultural products annually, including nearly 50% of the banana supply of the United States (*6*). Therefore, agricultural workers in Guatemala and similar LMICs are arguably essential not only for local food security but also for the food security of international trading partners, such as the United States.

In addition to increased risk for exposure to SARS-CoV-2, the virus that causes COVID-19, persons working in the agricultural sector might also be at increased risk for poor clinical outcomes from COVID-19 because of a high prevalence of comorbidities associated with environmental stress, such as chronic kidney disease of unknown origin (Mesoamerican nephropathy) (*7*–*9*). Economic outcomes, such as work absenteeism and decreased job performance while working (presenteeism), are also critical factors, as is the case with influenza (*10*–*14*). Because agricultural workers are often the primary income earners for their households, the consequences may extend to their households and communities. Despite the increased clinical and economic vulnerability of agricultural workers and their critical role in global food security, little is known about the socioeconomic consequences of COVID-19 and other respiratory illnesses among this essential workforce, and the subsequent effects on their households and communities.

The Agricultural Workers and Respiratory Illness Impact (AGRI) Study was designed as an influenza cohort and expanded to include other viral respiratory pathogens, including SARS-CoV-2. The study has 2 primary aims: to characterize the clinical and socioeconomic outcomes of acute respiratory viral infections among farm workers in Guatemala, and to measure the effectiveness of a workplace-based vaccination program in improving these outcomes. Here, we provide a comprehensive description of the AGRI cohort and a summary of clinical and economic outcomes from the first year of virologic surveillance.

## Methods

### Study Setting and Population

This 5-year study was conducted within a large banana farm in the coastal lowlands of southwestern Guatemala. Farm workers are exposed to high temperatures and humidity and are at risk for environment-associated chronic medical conditions, such as chronic kidney disease of unknown origin (*7*,*15*). Previous surveys (2015, 2017–2018) found a predominantly young, male, and economically vulnerable workforce. Farmworkers are typically the sole income earners for their households and report high rates of food insecurity, similar to other agribusiness workers in the region and migrant worker populations in the United States (*16*,*17*). The regional population experiences high levels of food insecurity, stunting, poverty, and communicable diseases and low access to healthcare (*18*,*19*).

As is typical in many agribusinesses, field workers and packaging workers receive baseline pay plus daily bonuses based on productivity recorded by the company. Managers and workers in administrative job categories are paid by day. Workers who become ill and receive excused absences from their managers receive two thirds of baseline pay for the duration of the excused absence, up to a maximum of US $15.60/day. Workers with laboratory-confirmed SARS-CoV-2 are mandated to quarantine at home with excused absences for up to 2 weeks.

All eligible workers within the 9 banana farm worksites were offered enrollment in the study during June 15‒December 30, 2020, and annually thereafter. Inclusion criteria were age >18 years, plans to remain employed by the agribusiness for >1 year, access to a telephone, and agreement to enable use of company-based absenteeism and job performance records. For this analysis, participant follow-up was performed through October 10, 2021; all study procedures (testing and follow-up) performed after that date were considered missing, even if the associated influenza-like illness (ILI) case was previously identified.

After written informed consent was obtained, study nurses collected contact information and demographic, occupational, socioeconomic, and clinical data, including risk factors for severe COVID-19. Workers provided enrollment and annual blood specimens that were screened for markers of chronic kidney disease (e.g., estimated glomerular filtration rate [*20*]), SARS-CoV-2 nucleocapsid IgG (Elecsys Immunoassay; Roche, https://www.roche.com), and in some instances SARS-CoV-2 neutralizing antibodies (Beckham/Santiago Laboratories, University of Colorado, Aurora, CO, USA). Workers leaving employment had exit interviews and were removed from the study, but data collected during their employment were retained in the study database.

### Surveillance for ILI

After enrollment, all workers began prospective active surveillance for ILI, initially defined as a self-reported fever/temperature >38°C and cough in the previous 10 days, to focus on detection of influenza (*21*). In January 2021, the ILI case definition was expanded to include fever, cough, or shortness of breath in the previous 10 days (COVID-19–like illness [*22*]), to increase sensitivity of COVID-19 case detection (*23*).

We used 3 strategies for detecting ILI. The first strategy was symptom screening through workers self-reporting symptoms to a study nurse during weekly worksite visits, work supervisors routinely querying workers for cough and fever at daily team meetings, and telephone contact to a study nurse by workers experiencing symptoms at any time. The second was sentinel surveillance of all workers who had ILI and presented to worker health posts within the farm. The third was active monitoring and ILI screening phone calls to absent workers identified on the company absenteeism registry. During worksite visits, study nurses visited worksites at given times each week, and workers were able to self-report to the nurse at that time. Work supervisors (or nurses at health posts) could also notify the study team on behalf of workers after obtaining their permission. In February 2021, absenteeism calls were discontinued because those ILI case-patients were consistently identified through other surveillance approaches.

### Syndromic Illness Characterization

Study nurses interviewed workers who had ILI and collected clinical, epidemiologic, and outcome data for the workers and general epidemiologic and socioeconomic outcome data for their households. Study nurses also collected nasopharyngeal swab specimens, which were placed in viral transport medium and tested within 24 hours for SARS-CoV-2 by using the Q COVID rapid antigen test (Q-NCOV-01G; Biosensor SD, https://www.sdbiosensor.com) (*24*). Aliquots were also tested for influenza A/B viruses and respiratory syncytial virus (RSV) by using the Cobas Liat Influenza A/B (and RSV) real-time reverse transcription PCR (RT-PCR) instrument (Roche) (*25*). Patients who had ILI and tested positive for SARS-CoV-2, influenza, or RSV are hereafter referred to as SARS-CoV-2‒positive ILI, influenza-positive ILI, or RSV-positive ILI, respectively. The first 40 available ILI specimens collected through April 2021 and shipped to the University of Colorado were also tested for an additional 15 respiratory pathogens by using the multiplex BioFire FilmArray RP2.1 assay (*26*). Viral testing results were shared with participants when available (usually within 24 hours) and weekly with the Guatemala Ministry of Health.

### Clinical and Socioeconomic Outcome Assessments

The study relied on a case‒cohort study design to measure self-reported clinical and socioeconomic outcomes. All persons in the overall cohort with ILI were considered to be case-patients. Each week, a subcohort of 15 enrolled workers who did not have ILI in the preceding 28 days were selected at random (≈5% of the cohort/month) as controls. Study nurses administered follow-up surveys over the telephone to case-patients at 1 and 4 weeks after their ILI visit. Controls were notified that they had been selected (day 0) and received the same surveys 1 and 4 weeks later; controls did not undergo diagnostic testing, and a control who had ILI develop during the 4-week follow-up was considered to be a case-patient at the time of illness.

Clinical and well-being outcomes were collected by using the Influenza Intensity and Impact Questionnaire (FluiiQ) Inventory (*27*), which is a validated Spanish-language outcome measure designed for clinical and epidemiologic outpatient studies of influenza and RSV. The inventory consists of 13 items for symptom severity, a combined systemic score (7 items) and respiratory score (6 items). The well-being scores are impact on daily activities score (7 items), impact on emotions score (4 items), and impact on others score (5 items). Each combined score is averaged by the number of individual items such that all scores are 0‒3; a higher score indicated greater severity or negative impact on well-being. The follow-up surveys also collected health-seeking behavior (e.g., hospitalization, medication use).

During follow-up surveys, economic outcomes were assessed by using questions adapted from the 2016 World Health Organization Manual for Estimating the Economic Burden of Seasonal Influenza (*28*) and supplemented with the World Bank National Survey of Living Conditions (*29*), which includes a Spanish translation (La Encuesta Nacional de Condiciones de Vida) used in Guatemala (*30*). The survey collected data on direct medical costs, direct nonmedical costs (i.e., transportation), and indirect costs related to loss of productivity (i.e., absenteeism) for the worker and the household. Results were compared with the basic food basket price in Guatemala, which reflects the minimum kilocalories intake (2,262 kilocalories) for a 4.77-member household for 1 month (US $386.30 in March 2021) (*31*). Although not included in this analysis, company-reported individual-level data were linked to workers, including absenteeism, productivity metrics (task-specific units of production, such as tons of bananas harvested per day), and wages.

### Statistical Analysis

We calculated incidence density (number of cases per person-time of follow-up) of ILI and pathogen-specific ILI. We used descriptive statistics to calculate differences between clinical and socioeconomic outcomes between groups. For normally distributed continuous variables, we calculated means and SDs and used the Student *t*-test to determine major differences between groups. For non–normally distributed continuous variables, we calculated medians and interquartile ranges and used the Wilcoxon rank-sum test to determine major differences between groups. For categorical variables, we used χ^2^ and Fischer exact tests to determine major differences in distribution of categories between groups. For all analyses, p<0.05 was considered statistically significant.

### Ethics

The study was approved by the Colorado Multiple Institutional Review Board (protocol #19-1836) and the Guatemala Ministry of Health National Ethics Committee (HRMC-560-2020). The local Southwest Trifinio Community Advisory Board for Research agreed to the study. Workers receive no compensation for study participation.

## Results

**Figure 1:**
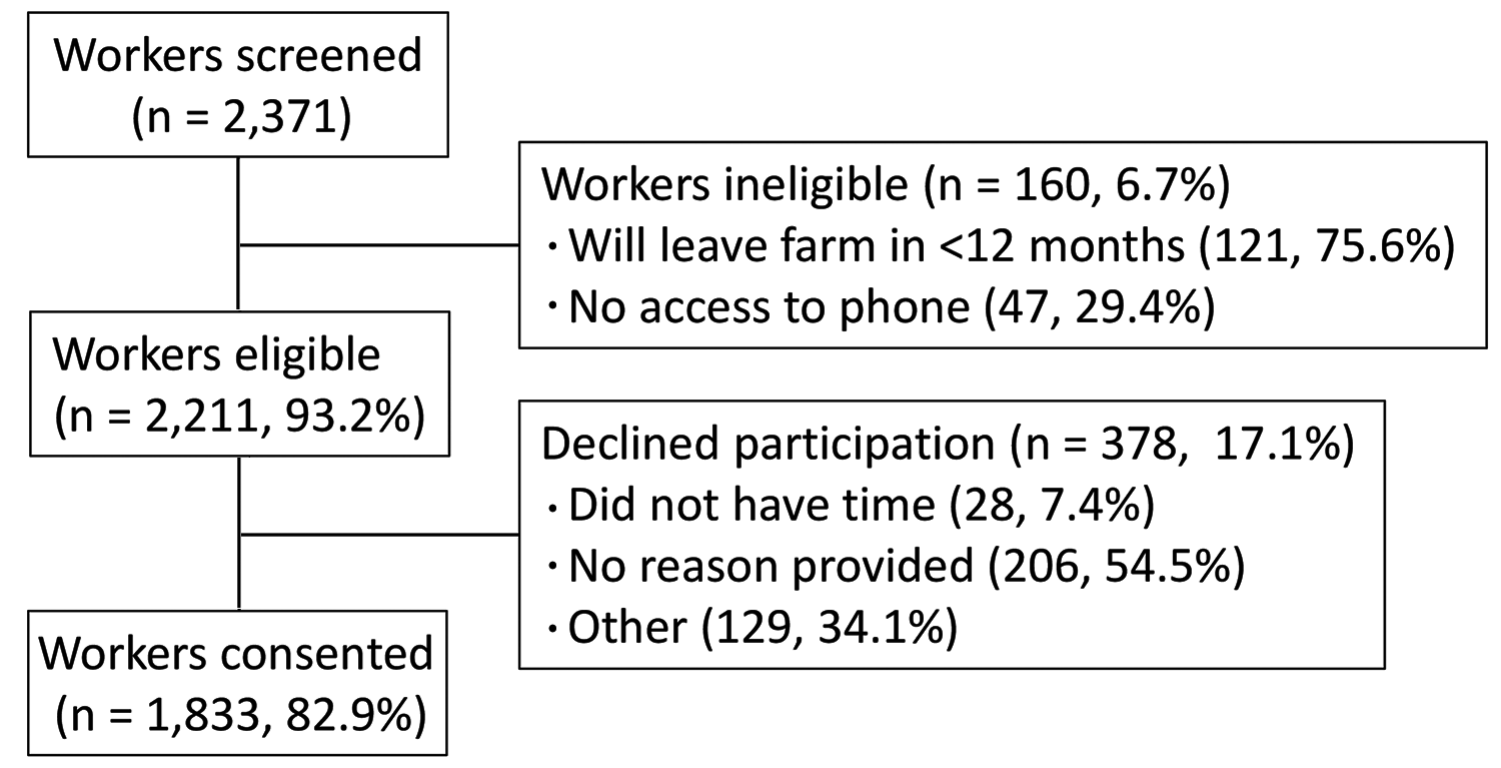
Flow diagram showing cohort of agricultural workers enrolled in the Agricultural Workers and Respiratory Illness Impact Study, southwestern Guatemala, June 2020‒October 2021, and followed through October 10, 2021. Ineligible and nonconsenting workers were able to provide multiple reasons for not participating. Only workers who completed the day 0 (diagnosis) visit were called on day 7 and day 28. Follow-up visits scheduled for after October 10, 2021, were considered missing.

During June 15, 2020‒October 10, 2021, a total of 2,371 workers were screened for enrollment; 160 (6.7%) were ineligible, and 378 (17.1%) declined participation (Figure 1). Of the 1,833 enrolled participants (Table 1), 1,590 (86.7%) remained active in the study as of October 10, 2021, representing 1,402.9 person-years of surveillance. Workers who declined participation were slightly younger than participants (29.6 vs. 30.9 years; p<0.01) but had similar sex distribution and ethnicity.

**Table 1:** Characteristics of 1,833 cohort participants in study of clinical and economic impact of COVID-19 in agricultural workers, Guatemala*

Characteristic	Value
Worker demographics	
Age, y, mean (SD)	30.9 (8.7)
Sex	
M	1,541 (84.1)
F	292 (15.9)
Latino ethnicity	801 (43.7)
Indigenous	113 (6.2)
Other	3 (0.2)
Do not know	916 (43.7)
Health worker	
Obesity, BMI >30 kg/m^2^, n = 1,159 with data	131 (11.3)
Class 1, 30‒-<35	103
Class 2, 35‒<40	24
Class 3, >40	4
Underlying conditions	
Kidney disease	58 (3.2)
Blood disorder, e.g., sickle cell disease	25 (1.4)
Cardiovascular disease, e.g., heart failure, CAD	29 (1.6)
Diabetes	27 (1.5)
Liver disease	19 (1.0)
Asthma	10 (0.6)
Pulmonary disease, e.g., COPD	10 (0.6)
Neurologic disease, e.g., stroke	10 (0.6)
Taking medications	234 (12.8)
Received influenza vaccine	108 (5.9)
Work conditions	
Type of work	
Administration	60 (3.3)
Field worker	1,264 (69.0)
Field manager	77 (4.2)
Packer/plant worker	413 (22.5)
Plant manager	19 (1.0)
Duration of employment, y	
<2	1,115 (60.9)
3‒4	242 (13.2)
>5	475 (25.9)
Monthly income, US$, median (IQR)	337.2 (311.3‒389.1)
Household conditions	
No. adults in house, median (IQR)	3 (2–4)
No. children in house, median (IQR)	2 (1–3)
Concern about food insecurity in last year	1,063 (58.0)
Household monthly income, US$, median (IQR)	363.2 (324.3­–505.8)
US$ spent in the past 7 days, median (IQR)	
Meat, fish, and seafood	25.9 (13.0–38.9)
Milk, eggs, and dairy products	15.6 (9.7–25.9)
Greens, vegetables, and fruit	13.0 (6.8–19.5)
Alcoholic drinks and tobacco	0 (0–0)

*Values are no. (%) except as indicated. BMI, body mass index; CAD, coronary artery disease; COPD, chronic obstructive pulmonary disease; IQR, interquartile range; US $, US dollars.

Most workers were male (84.1%) and worked in the fields (69.0%). Self-reported chronic medical conditions were uncommon except for obesity (body mass index >30 kg/m^2^, 11.3%) and kidney disease (3.2%); 12.8% of workers (n = 234) took medications, most of whom (n = 122, 52%) took vitamins, followed by pain relievers/anti-inflammatory drugs (14%), antimicrobial drugs (7%), diabetes-related medications (7%), and proton pump inhibitors (6%). Only 5.9% reported ever having received an influenza vaccination, including 17 (6.4%) of 267 workers who self-reported chronic diseases. Workers began to receive COVID-19 vaccination through the workplace in August 2021 (ChAdOx1, AstraZeneca, https://www.astrazeneca.com; and mRNA-1273, Moderna, https://www.modernatx.com). Of 1,334 workers enrolled during June‒December 2020 who had samples available, 616 (46.2%) were reactive for SARS-CoV-2 nucleocapsid IgG.

**Figure 2:**
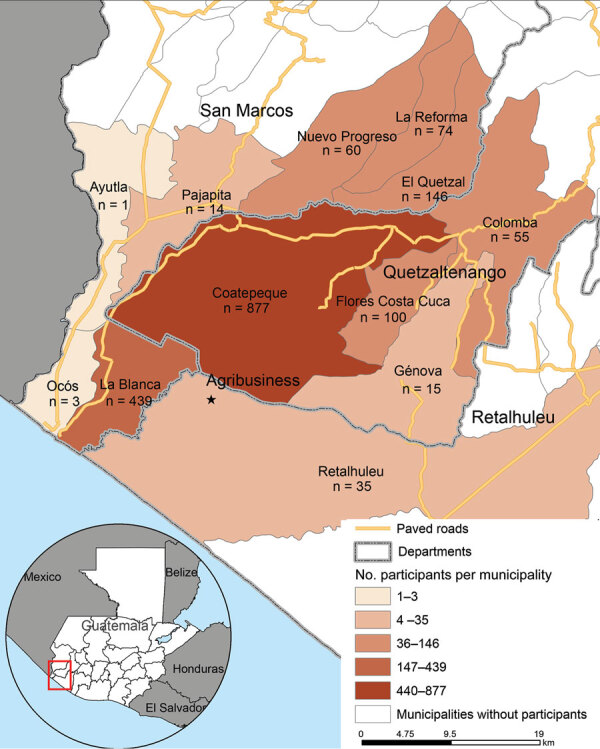
Study region (area 2,600 km^2^) for the Agricultural Workers and Respiratory Illness Impact Study, Guatemala, June 15, 2020‒October 10, 2021, showing number of enrolled agricultural workers living in each municipality. A total of 1,819 persons had reported data. Inset map shows location of study area in Guatemala.

Household size averaged 5.7 persons (3.3 adults, 2.3 children), and half the workers (n = 877; 48.2%) lived the urban municipality of Coatepeque; the study catchment area was ≈2,600 km^2^ (Figure 2). Median self-reported monthly income for the individual worker was US $337.20 (interquartile range $311.30‒$389.10) and for the household was US $363.20 (interquartile range $324.30‒$505.80); 58.0% of workers reported being worried about the inability to purchase food in the preceding 12 months.

### Asymptomatic Control Subjects

Of the 915 asymptomatic randomly selected controls (August 10, 2020–October 10, 2021), the study team was able to contact 696 (76.0%) by telephone. There were no significant differences in enrollment characteristics between those contacted and those not contacted. Of the 696 controls who were contacted initially, 623 (89.5%) were successfully contacted at 1 week and 588 (84.4%) at 4 weeks.

### Absenteeism

During August 31, 2020‒February 19, 2021, a total of 36 workers (51.4%) had >1 day of work absence. Study personnel contacted 504 (68.5%) after 3 attempts, and there were no differences between contacted and uncontacted workers other than number of children (2.7 vs. 2.2; p<0.01). We compiled risk associations for absenteeism (Appendix Table 1).

### Respiratory Illnesses

During June 15, 2020‒October 10, 2021, the study identified 169 ILI episodes occurring among 145 persons; of those, 136 (93.8%) persons (for 157 ILI episodes) completed the 7-day follow-up survey and 129 (89.0%) persons (for 149 ILI episodes) completed the 28-day follow-up survey by completion of analysis (Appendix Table 2). The mean (+SD) number of days of fever at the time of testing was 3.3 (+2.0) days and of cough was 3.3 (+1.9) days; 97.5% of samples were collected <7 days after symptom onset. Of the 153 ILI episodes (among 132 unique persons) who had completed SARS-CoV-2 antigen testing by completion of analysis, 43 (28.1%) were positive for SARS-CoV-2. Of 151 ILI episodes (among 131 persons) who had complete influenza and RSV RT-PCR testing, 6 (3.7%) were RSV positive and 0 were influenza positive.

**Figure 3:**
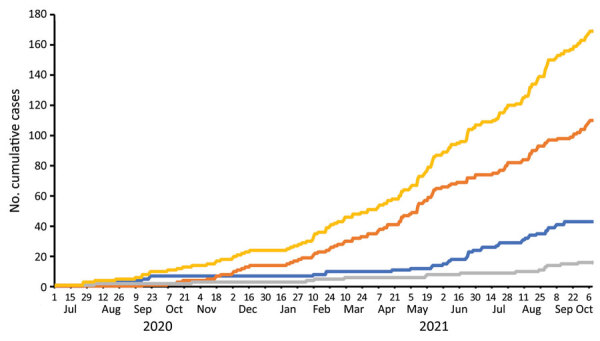
Cumulative influenza-like infections (ILI) among agricultural workers in the Agricultural Workers and Respiratory Illness Impact Study, Guatemala, June 15, 2020‒October 10, 2021. During June 2020–October 2021, ILI was defined as cough and fever. During January 2021, the ILI case definition was expanded to cough or fever or shortness of breath. Includes all-cause ILI (yellow), SARS-CoV-2‒positive ILI (blue), SARS-CoV-22‒negative‒ILI (orange), and ILI without testing obtained (gray).

Incidence density for ILI was 12.0/100 person-years and for SARS-CoV-2‒positive ILI was 3.1/100 person-years (Figure 3). The ILI and COVID-19 incidence densities for workers who were nucleocapsid IgG‒negative at enrollment (n = 718) were 7.1/100 person-years and 2.3/100 person-years, respectively. For workers who were positive for nucleocapsid IgG positive at enrollment (n = 616), ILI incidence density was 4.5/100 person-years and COVID-19 incidence density 0.4/100 person-years. Older age was associated with greater risk for SARS-CoV-2‒positive ILI compared with SARS-CoV-2‒negative ILI (mean 35.0 years vs. 29.6 years; p = 0.001); there was no significant difference by sex, presence of any comorbidity, or obesity.

BioFire FilmArray RP2.1 testing (n = 40) on available specimens confirmed 9 of 9 SARS-CoV-2 infections tested and 1 of 1 RSV infection and identified an additional 8 picornaviruses (rhinovirus/enterovirus target on FilmArray) and 6 seasonal coronavirus (3 NL63, 1 OC43, and 2 N229E) ILI cases. The adult worker was usually the index case-pateint within the household for SARS-CoV-2 ILI (>80%) infections (Table 2) and ILI (>85%) (Appendix Table 2).

**Table 2:** Comparison of self-reported outcomes in SARS-CoV-2‒positive ILI vs. SARs-CoV-2‒negative ILI in study of clinical and economic impact of COVID-19 in agricultural workers, Guatemala*

Characteristic	Day 0				Day 7				Day 28		
	SARS-CoV-2 positive, n = 43	SARS-CoV-2 negative, n = 110	p value†		SARS-CoV-2 positive, n = 41	SARS-CoV-2 negative, n = 103	p value†		SARS-CoV-2 positive, n = 38	SARS-CoV-2 negative, n = 97	p value†
Clinical symptoms											
No. days of fever, mean (SD)	3.3 (2.0)	2.3 (1.5)	<0.01		4.4 (2.8)	3.3 (2.5)	0.08		0.5 (0.0)	3.4 (2.2)	0.04
No. days of cough, mean (SD)	3.3 (1.9)	3.6 (2.1)	0.45		6.1 (2.3)	5.1 (2.6)	0.07		4.5 (4.9)	5.1 (3.5)	0.84
Had in the past 24 h											
Fever	32 (74.4)	60 (54.6)	0.02		2 (4.9)	11 (10.8)	0.35		4 (10.5)	4 (4.2)	0.22
Nasal congestion	18 (41.9)	39 (35.5)	0.46		5 (12.2)	17 (16.7)	0.50		2 (5.3)	6 (6.3)	1.00
Myalgia	23 (53.5)	46 (41.8)	0.19		3 (7.3)	10 (9.8)	0.76		5 (13.2)	6 (6.3)	0.19
Headache	30 (69.8)	55 (50.0)	0.03		13 (31.7)	21 (20.6)	0.16		8 (21.1)	14 (14.6)	0.36
Cough	24 (55.8)	39 (35.5)	0.02		7 (17.5)	14 (13.7)	0.57		3 (7.9)	7 (7.3)	1.00
Sore throat	15 (34.9)	50 (45.5)	0.23		6 (14.6)	14 (13.7)	0.89		4 (10.5)	7 (7.3)	0.51
Dysgeusia	21 (48.8)	27 (24.6)	<0.01		8 (19.5)	6 (5.9)	0.01		3 (7.9)	2 (2.1)	0.14
Expectoration	16 (37.2)	45 (40.9)	0.67		12 (29.3)	18 (17.7)	0.12		7 (10.5)	8 (8.3)	0.74
Fatigue	24 (55.8)	30 (27.3)	<0.01		7 (17.1)	11 (10.8)	0.31		6 (15.8)	5 (5.2)	0.04
Anosmia	19 (44.2)	19 (17.3)	<0.01		9 (22.0)	3 (2.9)	<0.01		3 (7.9)	2 (2.1)	0.14
Loss of appetite	17 (39.5)	31 (28.2)	0.17		4 (9.8)	5 (4.9)	0.28		1 (2.6)	2 (2.1)	1.00
Dyspnea	13 (30.2)	25 (22.9)	0.35		4 (9.8)	5 (4.9)	0.28		4 (10.8)	1 (1.0)	0.02
Neck pain	14 (33.3)	18 (16.4)	0.02		6 (14.6)	5 (4.9)	0.05		4 (10.5)	4 (4.2)	0.22
Interrupted sleep	11 (25.6)	19 (17.3)	0.24		8 (19.5)	6 (5.9)	0.01		3 (7.9)	4 (4.2)	0.40
Wheezing	4 (9.5)	14 (12.7)	0.78		1 (2.4)	5 (4.9)	0.67		4 (10.5)	1 (1.0)	0.02
Well-being‡											
Had difficulty in past 24 h											
Getting out of bed	16 (37.2)	26 (23.6)	0.09		5 (12.2)	4 (3.9)	0.12		2 (5.3)	4 (4.2)	1.00
Preparing meals	8 (18.6)	10 (9.1)	0.10		3 (7.3)	1 (1.0)	0.07		2 (5.3)	2 (2.1)	0.32
Performing usual tasks…	16 (37.2)	27 (24.6)	0.12		4 (9.8)	3 (2.9)	0.10		3 (7.9)	3 (3.1)	0.35
Leaving the home	13 (30.2)	14 (12.7)	0.01		6 (14.6)	0	<0.01		2 (5.3)	2 (2.1)	0.32
Concentrating	18 (41.9)	23 (20.9)	0.01		4 (9.8)	2 (2.0)	0.06		2 (5.3)	5 (5.2)	1.00
Taking care of things	16 (37.2)	13 (11.7)	<0.01		4 (9.8)	0	0.01		2 (5.3)	4 (4.2)	1.00
Leaving the room	9 (20.9)	14 (12.7)	0.20		4 (9.7)	0	0.01		2 (5.3)	2 (2.1)	0.32
Had in past 24 h											
Irritable	25 (58.1)	37 (33.6)	0.01		4 (9.8)	8 (7.8)	0.74		4 (10.5)	6 (6.3)	0.47
Defenseless	18 (41.9)	20 (18.2)	<0.01		5 (12.2)	5 (4.9)	0.12		3 (7.9)	3 (3.1)	0.35
Worried	19 (44.2)	35 (31.8)	0.15		13 (31.7)	8 (7.8)	<0.01		5 (13.2)	10 (10.4)	0.65
Frustrated	15 (34.9)	16 (14.6)	<0.01		3 (7.3)	6 (5.9)	0.72		2 (5.3)	5 (5.2)	1.00
People worrying	30 (69.8)	57 (51.8)	0.04		18 (43.9)	21 (20.6)	<0.01		11 (29.0)	18 (18.8)	0.20
Being a burden	13 (30.2)	30 (27.3)	0.71		9 (22.0)	10 (9.8)	0.05		5 (13.2)	9 (9.4)	0.52
People being a burden	13 (30.2)	24 (21.8)	0.27		8 (19.5)	8 (7.8)	0.05		7 (18.4)	7 (8.3)	0.10
Dependent	16 (37.2)	23 (20.9)	0.04		10 (24.4)	9 (8.8)	0.01		6 (15.8)	10 (10.4)	0.39
People have to do extra things	15 (34.9)	21 (19.1)	0.04		10 (24.4)	10 (9.8)	0.02		8 (21.1)	8 (8.3)	0.04
FluiiQ severity scores§											
Systemic score	0.87 (0.68)	0.52 (0.55)	<0.01		0.21 (0.35)	0.12 (0.24)	0.19		0.13 (0.31)	0.09 (0.28)	0.41
Respiratory score	0.50 (0.43)	0.47 (0.45)	0.64		0.16 (0.28)	0.16 (0.32)	0.99		0.14 (0.4)///	0.08 (0.21)	0.41
Impact on daily activities	0.50 (0.85)	0.22 (0.40)	0.01		0.14 (0.40)	0.02 (0.08)	0.05		0.06 (0.21)	0.04 (0.20)	0.71
Impact on emotions score	0.62 (0.57)	0.31 (0.45)	<0.01		0.17 (0.34)	0.07 (0.22)	0.09		0.20 (0.24)	0.08 (0.23)	0.60
Impact on others score	0.65 (0.65)	0.44 (0.64)	0.08		0.37 (0.52)	0.16 (0.40)	0.03		0.25 (0.47)	0.16 (0.41)	0.27
Epidemiology											
No. in house with similar illness in past 2 wks	0.30 (0.64)	0.13 (0.36)	0.10		0.34 (0.62)	0.17 (0.49)	0.09		0.11 (0.39)	0.14 (0.41)	0.61
Index case in house, no. (%)											
Self	35 (81.4)	97 (88.2)	0.13		34 (82.9)	96 (94.1)	0.10		33 (86.8)	90 (93.8)	0.24
Spouse	2 (4.7)	3 (2.7)			1 (2.4)	1 (1.0)			1 (2.6)	1 (1.0)	
Parent	0	2 (1.8)			1 (2.4)	1 (1.0)			0	0	
Sibling	3 (7.0)	3 (2.7)			3 (7.3)	2 (2.0)			3 (7.9)	3 (3.1)	
Cousin	1 (2.3)	0			1 (2.4)	0			0	0	
Child	0	4 (3.6)			0	1 (1.0)			0	2 (2.1)	

*Values are no. (%) except as indicated. ILI, influenza-like illness. †p values are for t-tests for continuous variables and χ^2^ test for categorical variables. ‡Items truncated per FluiiQ licensing agreement; full items available from the author. §Higher scores indicate worse outcomes.

**Figure 4:**
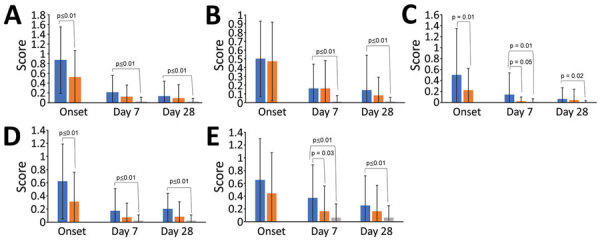
FluiiQ severity scores for agricultural workers in the Agricultural Workers and Respiratory Illness Impact Study, Guatemala, June 15, 2020‒October 10, 2021. Scores (range 0–3), by subdomain, are shown for workers who had SARS-CoV-2‒positive influenza-like illness (ILI), SARS-CoV-2‒negative ILI, and asymptomatic controls. Higher score indicates greater clinical severity (A, B) or greater negative impact on well-being (C, D, E). A) Systemic score; B) respiratory score; C) impact on daily activities; D) impact on emotions; E) impact on others. Significant differences (p<0.05) are identified within each group. Blue indicates SARS-CoV-2‒positive ILI, orange indicates SARS-CoV-2‒negative ILI, and gray indicates asymptomatic control subjects. Error bars indicate means and SDs.

Workers who had SARS-CoV-2‒positive ILI had longer fever duration at the time of diagnosis (day 0; 3.3 days vs. 2.3 days; p<0.01) and increased frequency of anosmia (44.2% vs. 17.3%; p<0.01) and dysgeusia (48.8% vs. 24.6%; p<0.01), compared with SARS-CoV-2‒negative workers (Table 2). SARS-CoV-2 case-patients were also more likely to have difficulty concentrating (41.9% vs. 20.9%; p = 0.01), irritability (58.1% vs. 33.6%; p = 0.01), and dependence on others (37.2% vs. 20.9%; p = 0.04). SARS-CoV-2‒positive workers had higher systemic FluiiQ severity scores (indicating greater disease severity) at diagnosis than did SARS-CoV-2‒negative workers, but other differences in clinical scores remained nonsignificant. SARS-CoV-2‒positive workers reported worse impact scores for daily activities (0.50 vs. 0.22; p = 0.01) and emotions (0.62 vs. 0.31; p<0.01) than for SARS-CoV-2‒negative workers at diagnosis and worse impact on others score at day 7 (0.37 vs. 0.16; p = 0.03), but all other FluiiQ well-being scores showing a similar nonsignificant trend (Table 2; Figure 4). Among ILI cases, we found no significant difference in FluiiQ score based on age, sex, and presence of any comorbidity or obesity. We compiled clinical outcomes of workers who had ILI episodes versus asymptomatic controls (Appendix Table 2).

### Economic Outcomes

**Figure 5:**
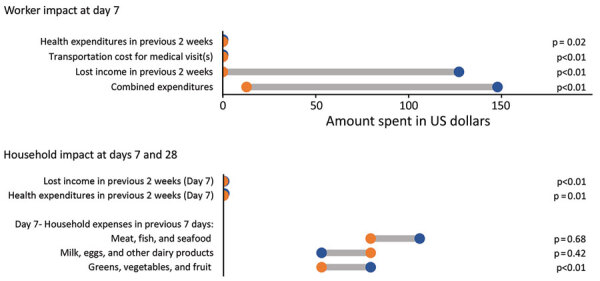
Differences in expenditures between SARS-CoV-2‒positive and SARS-CoV2‒negative agricultural workers who had influenza-like illness (ILI) in the Agricultural Workers and Respiratory Illness Impact Study, Guatemala, June 15, 2020‒October 10, 2021. Workers who had SARS-CoV-2‒positive ILI (dark blue circle) reported greater lost income and combined expenditures related to their illnesses in the week after their illness than SARS-CoV-2‒negative workers who had ILI (orange circle).

Compared with SARS-CoV-2‒negative workers who had ILI, SARS-CoV-2‒positive workers had greater self-reported lost income (median US $127.10 vs. $0; p<0.01), and combined (healthcare, transportation, lost wages) total cost (US $147.90 vs. US $12.70; p<0.01) at day 7 (reported over the preceding 2 weeks) (Figure 5). Workers infected with SARS-CoV-2 also had more days of work absence (p<0.01); most (81.8%) had >5 days of work absence. Household expenditures on fruits/vegetables were higher at day 7 for SARS-CoV-2‒positive workers vs. SARS-CoV-2‒negative workers who had ILI (US $19.50 vs. US $13.00; p< 0.01). Differences for all other household expenditures between SARS-CoV-2 test-positive and test-negative workers were not statistically significant.

## Discussion

As of October 10 2021, farm workers in Guatemala in this prospective cohort study experienced a substantial burden of acute respiratory illness during the COVID-19 pandemic, of which 1/4 tested positive for SARS-CoV-2; those with COVID-19 had greater disease severity, absenteeism, and economic losses than workers with SARS-CoV-2‒negative ILI. Similar to limited data for the United States (*1*), farm workers in Guatemala were at risk for SARS-CoV-2 infection (3.1 cases/100 person-years) throughout 2020–2021. Nucleocapsid IgG at enrollment was protective against subsequent disease; additional analyses will explore this observation. Compared with other members of their households, the agricultural workers nearly always had the index symptomatic case. These findings, along with the critical role agricultural workers play in Guatemala and global food security (*4*,*6*), lend support to the prioritization of vaccinating agricultural workers against COVID-19.

Although preliminary, our findings suggest COVID-19 illness was associated with greater overall clinical severity and impairment, which persisted at 7-day and 28-day after illness, than for non‒SARS-CoV-2 ILI cases. COVID-19 symptoms were consistent with those reported elsewhere; higher frequencies of anosmia and dysgeusia and prolonged fever differentiating COVID-19 from other ILI cases. COVID-19 was strongly associated with irritability and difficulty concentrating, consistent with postacute sequelae of SARS-CoV-2 (long COVID) (*32*,*33*). The irritability and inability to concentrate, which persisted in some workers at both 7 and 28 days, might place workers at risk (e.g., when using machetes to harvest bananas and when operating heavy equipment). The FluiiQ well-being scores, which include socioemotional and functional activities, generally indicated more severe illness among workers who had COVID-19 compared with workers who had another ILI at the time of diagnosis and day 7; the trend was nonsignificant at 28 days. It is unknown to what extent symptoms or sequelae persist beyond 28 days in this population.

Agricultural workers in this cohort experienced a major economic impact from COVID-19. Self-reported data suggest a major difference in absenteeism, lost earnings, and total costs between COVID-19 and other ILI cases. Median monthly household income (US $363.20), already just below the mean basic monthly food basket price in Guatemala (US $386.30), was reduced greatly for workers who had COVID-19 (median lost income US $127.10, median total cost of illness US $147.90), placing these households at increased risk for food insecurity and economic hardship. Economic insecurity is one of the primary drivers of emigration from Guatemala (*34*,*35*); thus, the economic impact and policy implications of COVID-19 on these agricultural workers and their households, as well as others in similar settings, might extend beyond the borders of Guatemala.

Although SARS-CoV-2 was the most frequently detected respiratory pathogen among workers who had ILI, we detected no cases of influenza and only 6 cases of infection with RSV. Influenza and RSV circulate year-round in Guatemala and comprise a substantial proportion of ILI cases in population-based studies in Central and South America (*36*–*39*). The lower incidence observed in our cohort suggested mitigation strategies (primarily closing of schools, mask use, and some level of physical distancing) might have been effective in limiting some transmission of influenza and RSV. The observation cases of rhinovirus/enterovirus and seasonal coronaviruses (NL63, OC43, and N229E) in a subset of our cohort is consistent with other reports (*40*,*41*), although the reasons for these detections despite physical distancing measures merit further study. The AGRI cohort and similar studies will provide useful observations on the effectiveness of population-based preventive measures, such as vaccines, on the burden of respiratory pathogens. Also, our data demonstrated that syndromic surveillance in the workplace is a feasible population-based approach to rapidly characterize an emerging pathogen.

The AGRI study design had some inherent strengths and limitations. Although the study included weekly visits to worksites to identify symptomatic ILI case-patients, it still required some level of self-reporting to study personnel, and therefore might underestimate incidence. Workers with laboratory-confirmed SARS-CoV-2 are required to isolate and might be incentivized to underreport illness to avoid lost wages, thus providing a bias toward lower incidence and more severe cases of disease being reported. Required isolation probably increased duration of absenteeism for workers who are SARS-CoV-2 positive, although it still reflected the consequences of COVID-19 in this population. Self-reported study outcomes are also subject to recall bias, which we aimed to minimize by including control subjects who had similar follow-up. Laboratory test results are provided to the worker when available; thus self-reported outcomes might be impacted by diagnostic bias.

We did not perform pathogen testing on controls. We used an antigen test for detection of SARS-CoV-2 infection and an ELISA for detection of nucleocapsid IgG, which might have decreased performance compared with PCR and virus neutralization assays, respectively. Testing was nearly always (>97%) performed within 7 days of symptom onset, and future studies will compare various testing approaches. Future studies will also include company-reported data, which will provide a more objective assessment of wages, enabling us to compare self-reported and company-reported metrics. Finally, to decrease the risk for healthy worker bias (*42*), the study collected postacute (28-day) outcomes on all ILI case-patients and will ultimately measure loss of employment (using company data) as an outcome measure of ILI.

In conclusion, preliminary data from the AGRI cohort suggest major clinical and socioeconomic impacts of respiratory illnesses, especially COVID-19, on agricultural workers in Guatemala. The study demonstrates the feasibility and value of conducting workforce-based syndromic surveillance during epidemic activity and uses several innovative approaches to measure disease outcomes in acute and postacute settings, such as active surveillance and molecular diagnostics within a large banana farm and company-reported economic measures. It also provides a more comprehensive assessment of how communicable diseases economically effect an essential, yet vulnerable, workforce population and their households. Given the high clinical and economic burden of COVID-19 among agricultural workers, and their probable role in household transmission of COVID-19, our results support prioritizing persons working in the agricultural sector for vaccination against COVID-19, potentially through the workplace.

Appendix Additional information on clinical and economic impact of COVID-19 on agricultural workers, Guatemala.
