# Supplementary material for: Clinical and Economic Impact of COVID-19 on Agricultural Workers, Guatemala
Source: Emerg Infect Dis. 2022 Dec;28(Suppl 1):S277–87. doi: 10.3201/eid2813.212303 (PMC9745239; doi:10.3201/eid2813.212303)
Supplement: Appendix — Additional information on clinical and economic impact of COVID-19 on agricultural workers, Guatemala. [file 21-2303-Techapp-s1.pdf]

Article DOI: https://doi.org/10.3201/eid2813.212303

# Clinical and Economic Impact of COVID-19 in Agricultural Workers, Guatemala

## Appendix.

**Appendix Table 1.** Absenteeism characteristics for agricultural workers, Guatemala, August 31, 2020–February 19, 2021*

| Characteristic | Workers with no absence, n = 695 | Workers with any absence, n = 736 | p value† |
|---|---|---|---|
| Worker demographics | | | |
| Age y, mean (SD) | 32.7 (9.1) | 30.0 (8.0) | <0.01 |
| Sex, no. (%) | | | |
| M | 611 (87.9) | 593 (80.6) | <0.01 |
| F | 84 (12.1) | 143 (19.4) | |
| Ladino ethnicity, no. (%) | 312 (44.9) | 315 (42.8) | 0.82 |
| Indigenous | 47 (6.8) | 50 (6.8) | |
| Other | 1 (0.1) | 2 (0.3) | |
| Do not know | 335 (48.2) | 369 (50.1) | |
| Worker health, no. (%) | | | |
| Asthma | 1 (0.1) | 7 (1.0) | 0.04 |
| Pulmonary disease | 4 (0.6) | 5 (0.7) | 0.80 |
| Kidney disease | 27 (3.9) | 22 (3.0) | 0.35 |
| Cardiovascular disease | 10 (1.4) | 10 (1.4) | 0.90 |
| Diabetes | 13 (1.9) | 7 (1.0) | 0.14 |
| Blood disorder (sickle cell disease) | 8 (1.2) | 14 (1.9) | 0.25 |
| Neurologic disease (stroke) | 3 (0.4) | 5 (0.7) | 0.53 |
| Liver disease | 5 (0.7) | 10 (1.4) | 0.24 |
| Obesity (BMI $\geq$30 kg/m$^2$), n = 773 | 67 (15.6) | 15 (4.4) | <0.01 |
| Taking medications | 83 (11.9) | 95 (12.9) | 0.57 |
| Ever received influenza vaccine | 50 (7.2) | 41 (5.6) | 0.21 |
| Work conditions | | | |
| No. absences/person, median (IQR) | 0 (0–0) | 5.3 (2–7) | <0.01 |
| Type of work, no. (%) | | | <0.01 |
| Administration | 43 (6.2) | 0 (0) | |
| Field worker | 42 (66.5) | 528 (71.7) | |
| Field manager | 54 (7.8) | 3 (0.4) | |
| Packer (factory worker) | 120 (17.3) | 205 (27.9) | |
| Factory manager | 16 (2.3) | 0 (0) | |
| How long worked at farm, y, no. (%) | | | <0.01 |
| $\leq$2 | 346 (49.8) | 453 (61.5) | |
| 3–4 | 104 (14.9) | 113 (15.4) | |
| $\geq$5 | 245 (35.3) | 170 (23.1) | |
| Monthly income, $USD, median (IQR) | 337.2 (311.3–389.1) | 337.2 (311.3–363.2) | <0.01 |
| Household conditions, mean (SD) | | | |
| No. adults in house | 3.2 (1.6) | 3.4 (1.8) | 0.01 |
| No. children in house | 2.3 (1.6) | 2.5 (1.7) | <0.01 |
| Concern about food insecurity in past year, no. (%) | 401 (57.7) | 464 (63.0) | 0.04 |
| Lacked money for food in the past 12 months, no. (%) | 506 (72.8) | 505 (68.6) | 0.08 |
| Household monthly income, $USD, median (IQR) | 363.2 (324.3–466.9) | 376.1 (324.3–466.9) | 0.06 |
| $USD spent in the past 7 d, median (IQR): | | | |
| Meat, fish, and seafood | 22.7 (13.0–32.4) | 19.5 (13.0–38.9) | 0.68 |
| Milk, eggs, and other dairy products | 13.0 (9.1–24.9) | 15.6 (9.7–25.9) | 0.07 |
| Greens, vegetables, and fruit | 13.0 (6.5–19.5) | 13.0 (6.5–19.5) | 0.83 |
| Alcoholic drinks and tobacco | 0 (0–0) | 0 (0–0) | 0.16 |

*BMI, body mass index; IQR, interquartile range; $USD, US dollars.
†By t-test for continuous variables and by $\chi^2$ test for categorical variables.

**Appendix Table 2.** Comparison of self-reported outcomes for agricultural workers who had ILI versus asymptomatic control subjects, Guatemala*

| Characteristic | Day 0 ILI, n = 169 | Day 7 ILI, n = 157 | Day 7 Control, n = 623 | p-value† | Day 28 ILI, n = 149 | Day 28 Control, n = 588 | p value† |
|---|---|---|---|---|---|---|---|
| Clinical symptom, mean (SD) | | | | | | | |
| No. days of cough | 3.5 (2.1) | 5.3 (2.6) | 3.4 (2.6) | 0.05 | 5.0 (3.5) | 3.7 (2.0) | 0.32 |
| No. days of fever | 2.6 (1.8) | 3.6 (2.6) | 1.5 (0.6) | <0.01 | 2.6 (2.3) | 3.5 (3.0) | 0.55 |
| Have you felt the following (past 24 h), no. (%) | | | | | | | |
| Fever | 96 (57.5) | 14 (9.0) | 4 (0.7) | <0.01 | 8 (5.4) | 3 (0.5) | <0.01 |
| Nasal congestion | 60 (35.9) | 23 (14.7) | 16 (2.7) | <0.01 | 9 (6.1) | 6 (1.1) | <0.01 |
| Myalgia | 73 (43.7) | 14 (9.0) | 23 (3.9) | 0.01 | 11 (7.5) | 11 (2.0) | <0.01 |
| Headache | 89 (53.3) | 37 (23.7) | 22 (3.7) | <0.01 | 25 (17.0) | 14 (2.5) | <0.01 |
| Cough | 69 (41.3) | 22 (14.2) | 2 (0.34) | <0.01 | 10 (6.8) | 1 (0.2) | <0.01 |
| Sore throat | 66 (39.5) | 20 (12.8) | 7 (1.2) | <0.01 | 12 (8.2) | 10 (1.8) | <0.01 |
| Dysgeusia | 51 (30.5) | 15 (9.6) | 2 (0.3) | <0.01 | 6 (4.1) | 2 (0.4) | <0.01 |
| Expectoration | 62 (37.1) | 30 (19.2) | 4 (0.7) | <0.01 | 12 (8.2) | 2 (0.4) | <0.01 |
| Fatigue | 58 (34.7) | 19 (12.2) | 18 (3.0) | <0.01 | 11 (7.5) | 19 (3.4) | 0.03 |
| Anosmia | 40 (24.0) | 13 (8.3) | 1 (0.2) | <0.01 | 6 (4.1) | 2 (0.4) | <0.01 |
| Loss of appetite | 49 (29.3) | 10 (6.4) | 6 (1.0) | <0.01 | 3 (2.0) | 7 (1.3) | 0.44 |
| Dyspnea | 41 (24.7) | 10 (6.4) | 1 (0.2) | <0.01 | 5 (3.4) | 1 (0.2) | <0.01 |
| Neck pain | 34 (20.5) | 11 (7.1) | 8 (1.3) | <0.01 | 8 (5.4) | 8 (1.4) | <0.01 |
| Interrupted sleep | 33 (19.8) | 15 (9.6) | 7 (1.2) | <0.01 | 7 (4.8) | 6 (1.1) | <0.01 |
| Wheezing | 19 (11.5) | 6 (3.9) | 1 (0.2) | <0.01 | 5 (3.4) | 0 (0) | <0.01 |
| Well-being | | | | | | | |
| Have you had difficulty with (past 24 h), no. (%)‡ | | | | | | | |
| Getting out of bed | 44 (26.4) | 10 (6.4) | 7 (1.2) | <0.01 | 7 (4.8) | 6 (1.1) | <0.01 |
| Preparing meals | 18 (10.8) | 4 (2.6) | 3 (0.5) | 0.04 | 4 (2.7) | 1 (0.2) | 0.01 |
| Performing usual tasks | 45 (27.0) | 9 (5.8) | 4 (0.7) | <0.01 | 6 (4.1) | 2 (0.4) | <0.01 |
| Leaving the home | 27 (16.2) | 6 (3.9) | 2 (0.3) | <0.01 | 4 (2.7) | 1 (0.2) | 0.01 |
| Concentrating | 43 (25.8) | 9 (5.8) | 7 (1.2) | <0.01 | 7 (4.8) | 1 (0.2) | <0.01 |
| Taking care of things | 31 (18.6) | 4 (2.6) | 3 (0.5) | 0.04 | 6 (4.1) | 1 (0.2) | <0.01 |
| Leave the room | 23 (13.8) | 4 (2.6) | 2 (0.3) | 0.02 | 4 (2.7) | 0 (0) | <0.01 |
| Have you felt the following (past 24 h), no. (%) | | | | | | | |
| Irritable | 66 (39.5) | 13 (8.3) | 10 (1.7) | <0.01 | 10 (6.8) | 5 (0.9) | <0.01 |
| Defenseless | 40 (24.0) | 12 (7.7) | 9 (1.5) | <0.01 | 6 (4.1) | 4 (0.7) | 0.01 |
| Worried | 59 (35.3) | 23 (14.7) | 13 (2.2) | <0.01 | 15 (10.2) | 20 (3.6) | <0.01 |
| Frustrated | 34 (20.4) | 10 (6.4) | 7 (1.2) | <0.01 | 7 (4.8) | 8 (1.4) | 0.01 |
| People worrying | 93 (55.7) | 40 (25.6) | 56 (9.4) | <0.01 | 29 (19.7) | 69 (12.4) | 0.02 |
| Being a burden | 47 (28.1) | 20 (12.8) | 12 (2.0) | <0.01 | 14 (9.5) | 15 (2.7) | <0.01 |
| People being concerned | 38 (22.8) | 16 (10.3) | 16 (2.7) | <0.01 | 15 (10.2) | 15 (2.7) | 0.01 |
| Dependent | 42 (25.2) | 19 (12.2) | 20 (3.4) | <0.01 | 16 (10.9) | 19 (3.4) | <0.01 |
| People doing extra work | 37 (22.2) | 21 (13.5) | 22 (3.7) | <0.01 | 16 (10.9) | 20 (3.6) | <0.01 |
| Flu-iiQ Severity scores | | | | | | | |
| Systemic score | 0.60 (0.47) | 0.14 (0.28) | 0.02 (0.09) | <0.01 | 0.10 (0.28) | 0.02 (0.07) | <0.01 |
| Respiratory score | 0.46 (0.44) | 0.15 (0.11) | 0.01 (0.07) | <0.01 | 0.09 (0.27) | 0.01 (0.05) | <0.01 |
| Impact on daily activities | 0.28 (0.49) | 0.05 (0.22) | 0.01 (0.06) | 0.01 | 0.04 (0.19) | 0.004 (0.03) | 0.02 |
| Impact on emotions score | 0.39 (0.51) | 0.11 (0.28) | 0.02 (0.09) | <0.01 | 0.07 (0.22) | 0.02 (0.09) | <0.01 |
| Impact on others score | 0.49 (0.65) | 0.21 (0.43) | 0.06 (0.22) | <0.01 | 0.17 (0.41) | 0.06 (0.19) | <0.01 |
| Epidemiology | | | | | | | |
| No. in house with similar illness (past 2 weeks) | 0.17 (0.45) | 0.21 (0.52) | 0.01 (0.10) | <0.01 | 0.12 (0.38) | 0.01 (0.15) | <0.01 |
| Index case in house, no. (%) | | | | | | | |
| Self | 145 (86.8) | 142 (91.0) | 1 (14.3) | <0.01 | 135 (91.8) | 3 (42.9) | <0.01 |
| Spouse | 5 (3.0) | 2 (1.3) | 2 (28.6) | | 2 (1.4) | 1 (14.3) | |
| Parent | 2 (1.2) | 2 (1.3) | 1 (14.3) | | 1 (0.7) | 0 (0) | |
| Sibling | 6 (3.6) | 5 (3.2) | 0 (0) | | 6 (4.1) | 0 (0) | |
| Cousin | 1 (0.6) | 1 (0.6) | 0 (0) | | 0 (0) | 0 (0) | |
| Child | 4 (2.4) | 1 (0.6) | 3 (42.9) | | 2 (1.4) | 2 (28.6) | |

*ILI, influenza-like illness.
†By t-test for continuous variables and by $\chi^2$ test for categorical variables.
‡Items truncated per Flu-iiQ licensing agreement; full items available from the corresponding author.
